# Diagnostic yield and clinical utility of a comprehensive gene panel for hereditary tumor syndromes

**DOI:** 10.1186/s13053-018-0102-4

**Published:** 2019-01-23

**Authors:** Jonas Henn, Isabel Spier, Ronja S. Adam, Stefanie Holzapfel, Siegfried Uhlhaas, Katrin Kayser, Guido Plotz, Sophia Peters, Stefan Aretz

**Affiliations:** 1 Institute of Human Genetics, University of Bonn, Sigmund-Freud-Str. 25, D-53127 Bonn, Germany; 2 Center for Hereditary Tumor Syndromes, University of Bonn, Bonn, Germany; 3 Center for Experimental and Molecular Medicine, Cancer Center Amsterdam and Amsterdam Gastroenterology & Metabolism, Amsterdam UMC, University of Amsterdam, Amsterdam, The Netherlands; 4 Department of Internal Medicine I, University of Bonn, Bonn, Germany; 5 Department of Medicine I, Biomedical Research Laboratory, University of Frankfurt, Frankfurt, Germany

**Keywords:** Familial tumor syndromes, Hereditary cancer, Tumor predisposition syndromes, Targeted sequencing, Next generation sequencing

## Abstract

**Background:**

In a considerable number of patients with a suspected hereditary tumor syndrome (HTS), no underlying germline mutation is detected in the most likely affected genes. The present study aimed to establish and validate a large gene panel for HTS, and determine its diagnostic yield and clinical utility.

**Methods:**

The study cohort comprised 173 patients with suspected, but unexplained, HTS (group U) and 64 HTS patients with a broad spectrum of known germline mutations (group K). All patients in group U presented with early age at onset, multiple tumors, and/or a familial clustering of various tumor types; no germline mutation had been identified during routine diagnostics. Sequencing of leukocyte DNA was performed for the 94 HTS genes of the Illumina TruSight™Cancer Panel and 54 additional HTS genes.

**Results:**

The sensitivity of the panel to identify known germline variants was 99.6%. In addition to known mutations, a total of 192 rare, potentially pathogenic germline variants in 86 genes were identified. Neither the proportion of rare variants per patient (group K: 0.9 variants; group U: 0.8 variants) nor the proportion of variants in the most frequently mutated, moderately penetrant genes *CHEK2* and *ATM* showed significant inter-group difference. Four of the five patients from group U with a truncating *CHEK2* mutation had thyroid cancer, pointing to a broader tumor spectrum in patients with pathogenic *CHEK2* variants. In 22% of patients from group K, a further potential causative variant was identified. Here, the most interesting finding was an *NF1* nonsense mutation in a child with a known *TP53* frameshift mutation*.* In 17% of patients from group U, potential causative variants were identified. In three of these patients (2%), mutations in *PMS2*, *PTEN,* or *POLD1* were considered to be causative. In both groups, incidental findings with presumptive predictive value were generated.

**Conclusions:**

The gene panel identified the genetic cause in some prescreened, unexplained HTS patients and generated incidental findings. Some patients harbored predicted pathogenic mutations in more than one established HTS gene, rendering interpretation of the respective alterations challenging. Established moderate risk genes showed an almost equal distribution among patients with known and unexplained disease.

**Electronic supplementary material:**

The online version of this article (10.1186/s13053-018-0102-4) contains supplementary material, which is available to authorized users.

## Background

Monogenic inherited predisposition accounts for approximately 5–8% of solid malignancies, with high variability being observed between cancer types [1, 2]. A considerable fraction of monogenic hereditary tumor syndromes (HTS) are largely attributable to germline mutations in genes involved in cell cycle regulation, cellular proliferation, and DNA repair. To date, research has identified more than 100 cancer predisposing genes (CPG) [3]. Germline mutations in these genes represent the underlying cause of approximately 40 clinically distinct HTS, or contribute as moderately penetrant risk factors to a variety of benign and malignant tumors.

The precise identification and delineation of HTS is an important task of medical geneticists and other health care professionals, since mutation carriers have an increased lifetime risk for a largely syndrome-specific spectrum of malignancies. Prognosis in these patients can be decisively improved by early detection and treatment. This in turn can be achieved through established surveillance programs [4, 5]; prophylactic surgery; and tailored therapies, such as PARP inhibitors in patients with BRCA1/2 mutations or PD-1 blockade in patients with microsatellite-instable colorectal cancer [6]. These approaches represent successful examples of personalized medicine.

Until recently, however, screening for a pathogenic germline mutation required the time-, cost- and personnel-intensive examination of individual genes by conventional Sanger DNA sequencing. By this, investigation was restricted to a few individual genes and to high risk groups, which were selected according to the fulfillment of specific clinical criteria (early age at onset, multiple tumors, familial clustering of tumors). These clinical criteria were therefore introduced in order to ensure a high specificity and optimize the cost-benefit ratio. However, this approach often results in low sensitivity.

Although early age at onset, multiple primary tumors and familial clustering of tumors are common features of HTS, in a considerable number of families, the established diagnostic criteria for a hereditary form are not fulfilled or overlooked due to the broad phenotypic overlap that exists between many tumor syndromes and variable phenotypes. This results in inadequate surveillance and treatment. For example, in a study by LaDuca [7], around 30% of patients with suspected hereditary colorectal cancer in whom a pathogenic germline mutation was identified using a multi-gene panel did not meet the corresponding diagnostic criteria. Moreover, in many patients with a suspected HTS, no underlying germline mutation is identified in the genes suggested by the respective tumor spectrum of the patient and the relatives.

The implementation of next generation sequencing (NGS) as high-throughput, massive parallel sequencing method, facilitates accurate and prompt diagnosis, and thus improved disease classification and patient care, through the simultaneous mutation screening of a set of relevant genes (gene panels or multi-gene analysis). Compared to Sanger sequencing, NGS is both time and resource effective [8, 9].

To evaluate the diagnostic yield and clinical utility of a more comprehensive screening approach, the present authors established and validated an extensive panel of HTS genes. The panel was used to screen 173 patients with a variety of suspected HTS without known pathogenic germline mutations, and 64 HTS patients with a broad spectrum of known pathogenic germline mutations. The sensitivity of the panel was validated using the confirmed germline mutations and additional polymorphisms or variants of uncertain significance (VUS), identified in the 64 HTS cases during routine diagnostics. The cohort of patients with a known pathogenic mutation was also included to demonstrate the impact of additional rare variants in genetically explained cases in comparison to the unexplained cases.

## Patients and methods

### Patients / data collection

All 237 index patients had been referred to the Institute of Human Genetics in Bonn from within Germany for molecular genetic investigation of a suspected HTS. Details of the patients and data collection are presented in the online material. 218 patients (92%) were unrelated index patients.

The cohort of the present study comprised on the one hand 64 HTS index patients with a confirmed pathogenic germline mutation in an HTS gene (group K, for known mutation). These cases presented with a broad spectrum of 14 distinct HTS (Table 1A, Additional file 1: Table S1).

**Table 1:** Clinical characteristics of the total patient cohort (*n* = 237)

Phenotype group	No.	Sex (m / f)	Mean age diagnosis (range)
(A) Patients with known pathogenic mutation (group K)			
HNPCC / Lynch syndrome	16	9/7	43 (25–75)
Polyposis	25	15/10	41 (1–67)
Li-Fraumeni syndrome	5	3/2	17 (0–44)
Cowden syndrome	7	2/5	36 (1–46)
Hereditary diffuse gastric cancer	5	1/4	42 (22–58)
Others	6	2/4	35 (3–70)
All	64	32/32	39 (0–75)
(B) Patients with unknown cause (group U)			
Familial / early onset CRC	38	20/18	40 (17–78)
Polyposis	32	17/15	49 (7–73)
Li-Fraumeni-like syndrome	39	13/26	33 (1–77)
Cowden-like syndrome	20	2/18	44 (15–77)
Familial / early onset gastric cancer	4	1/3	36 (33–45)
Others	40	17/23	39 (0–71)
All	173	70/103	41 (0–78)

*CRC* = colorectal cancer, *f* = female, *HNPCC* = hereditary non-polyposis colorectal cancer; *m* = male

On the other hand 173 patients with a suspected, but genetically unexplained, HTS were included (group U, for previously unknown cause). All cases presented with an early age at onset, multiple primary tumors, and / or a striking familial clustering of various tumors and were grouped into six different phenotypic classes (Table 1B). However, no mutation in the most likely affected genes had been identified during routine diagnostics.

Prior to the present study and within the context of routine diagnostics, leukocyte DNA from almost all 237 patients was screened for germline mutations in the gene responsible for the suspected HTS and, if applicable, the most likely differential diagnoses. The number of genes investigated previously within the context of routine diagnostics are shown in the online methods and in Additional file 2: Figure S1.

### High-throughput targeted sequencing

Genomic DNA was extracted from peripheral EDTA-anticoagulated blood samples using the standard salting-out procedure. NGS targeted mutation screening was performed using the TruSight™Cancer Sequencing Panel (Illumina, San Diego). This customized commercial kit includes 94 well established genes for HTS. The panel was extended using the Illumina DesignStudio by the addition of a further 54 relevant HTS genes (Additional file 3: Table S2), based on literature search. In total, 2426 selected targets were sequenced based on 3939 probes, corresponding to a cumulative target length of 626,960 bp. Library preparation, target enrichment, and high-throughput sequencing were performed according to the manufacturer’s protocol. All samples were sequenced on an Illumina MiSeq sequencer. Alignment was perfomed using the software of the Illumina MiSeq sequencer or the *SeqPilot* software (JSI Medical Systems), based on hg19.

### Variant filtering and prioritization

Data analysis was performed using the *Cartagenia BENCHlab NGS* platform version 3.0.4 (Leuven, Belgium). The targeted sequencing data were filtered for high-quality variants (read depth ≥ 10 and call quality ≥30). Afterwards truncating (nonsense mutations, frameshift deletions/insertions and mutations at highly conserved splice sites) rare variants (minor allele frequency (MAF) ≤ 0.01 according to allele frequencies from *dbSNP*, the *1000 Genomes* database (TGP), and the *Exome Variant Server* (EVS)) were selected. Apparent missense variants were only included if they showed an MAF of ≤0.001 and were predicted to have a deleterious or damaging effect by at least two of three or three of four in silico analysis tools (*PolyPhen-2, MutationTaster, LRT,* and *SIFT*). Furthermore, rare (MAF of ≤0.001) synonymous / silent variants were included, if they are located in the first or last three bases of an exon. Splicing efficiencies of the normal and mutant sequences were calculated using the software Alamut, which refers to the splice prediction programs Human Splicing Finder; SpliceSiteFinder-like (SSF); MaxEntScan (MES); GeneSplicer; and NNSPLICE 0.9 from BDGP (the *Berkeley Drosophila Genome Project*). To exclude obvious sequencing artifacts, a detailed visual inspection of the variants was performed in a read browser (*SeqPilot* software, JSI Medical Systems). For the evaluation of the most interesting variants, additional databases were used, in particular the Human Gene Mutation Database (HGMD); Locus-specific mutation databases (LOVD); and the Genome Aggregation Database (gnomAD).

### Validation of variants by sanger sequencing

All relevant variants were validated by Sanger sequencing of the corresponding region using standard protocols. The results were analyzed with the *SeqPilot* software (JSI Medical Systems). To avoid pseudogene amplification, Sanger sequencing of *PMS2* was based on long-range PCR with *PMS2*-specific primers, as described elsewhere [10].

### Statistical analysis

Proportions were compared using Fisher’s exact test, based on the number of patients carrying variants. All reported *p* values are two-tailed.

## Results

**Fig. 1:**
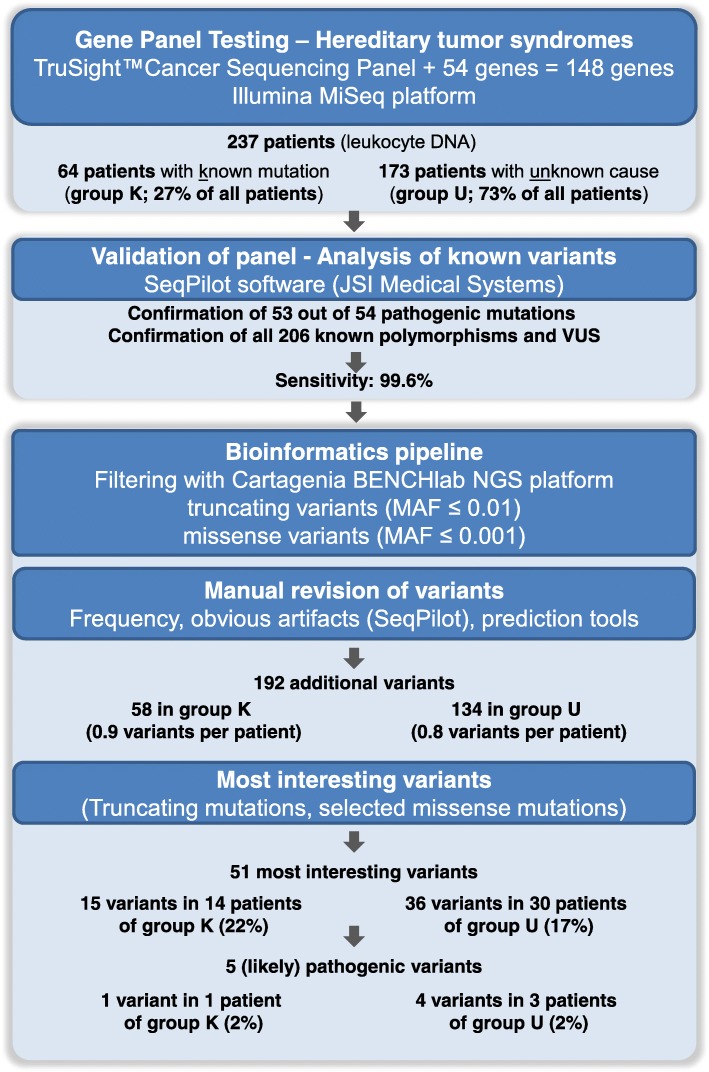
Flowchart showing each step of the analysis and the number of remaining variants in patients with known pathogenic mutation (Group K) and patients with unknown cause (Group U). MAF = minor allele frequency

A total of 237 patients were included: Group U comprised 173 patients with a suspected but unexplained HTS and group K comprised 64 patients with known germline mutations*.* The overall workflow of the study is shown in Fig. 1*.*

### Performance of targeted sequencing

A coverage of ≥10x, ≥20x, and ≥ 50x was achieved for 98.6, 97.4 and 93.0% of target bases, respectively. For the ≥30x threshold, visual inspection of the reads using the SeqPilot software revealed the following levels of exon coverage: (i) 59% of genes, all exons covered ≥30x; (ii) 35% of genes, several exons covered partially ≤30x; and (iii) 5% of genes, one or two whole exons covered ≤30x (five times exon 1 and five times exons other than exon 1) (Additional file 3: Table S2).

### Validation of the sensitivity of the gene panel

To assess the analytical sensitivity of the targeted sequencing approach in terms of detecting different types of genetic variation, blind evaluation was conducted of all germline variants - including mutations, benign variants, and VUS - that had been identified by Sanger sequencing in genes examined in both patient groups during routine diagnostics. These comprised 54 known mutations and 206 benign variants or VUS. Of the known mutations, 25 represented single base pair substitutions, and 29 represented insertions and/or deletions (including six deletions/duplications with a size of 14–65 bp and two indels encompassing five to 11 bp (Additional file 4: Table S3)). The NGS targeted sequencing panel detected 53 of the 54 (98%) known pathogenic germline mutations (Table 2). Two mutations were present in a homozygous state and two further mutations were present in a mosaic state (around 20% of reads). Moreover, the pipeline identified a frequent mutation in *MSH2* (c.942 + 3A > T), which is located within a large homopolymer. A 9 bp deletion in *STK11* was not validated due to low coverage (read depth = 1x). All 206 benign variants and VUS (mainly single base pair substitutions in heterozygous and homozygous state) were confirmed. Thus, the sensitivity of the gene panel to detect variants was 99.6%.

**Table 2:** Validation of the sensitivity of the multi-gene panel – analysis of known germline mutations, benign variants and VUS

Group of variants	Identified by Sanger sequencing	Validated in gene panel	Sensitivity (%)	No. of Patients
Known pathogenic mutations	54	53^a^	98	63
Benign variants and VUS	206	206	100	80
All	260	259	99.6	120

^a^ Non validated variant

*STK11*:c.907_915del9;p.Ile303_Gln305del (coverage = 1)

VUS = variant of uncertain significance

### Diagnostic yield

A total of 192 additional germline variants in 86 genes matched the filter criteria: 58 variants in 35 patients from group K; and 134 variants in 92 patients from group U (*p* = 0.8) (Additional file 5: Table S4, Table 3, Fig. 1). The proportion of (additional) rare variants was similar in both patient groups (0.9 variants per patient in group K and 0.8 variants per patient in group U). In group K and U, respectively, 45% or 47% of the patients carried no additional variant, while 47% or 47% carried 1–2 variants and 8% or 6% carried 3–4 variants (Additional file 2: Figure S2). After exclusion of the pathogenic variants (shown in bold in Table 4) a total of 187 variants must be classified currently as VUS, including 57 variants in 34 patients from group K (53%) and 130 variants in 88 patients from group U (51%).

**Table 3:** Distribution of additional rare variants and most interesting variants per patient group (Group K: Patients with known pathogenic mutation (*n* = 64); Group U: Patients with unknown cause (*n* = 173); all patients (*n* = 237))

	No. of variants (variants per patient)			No. of patients (%)			*p*-value
	Group K	Group U	All patients	Group K	Group U	All patients	
All additional variants	58 (0.9)	134 (0.8)	192 (0.8)	35 (55%)	90 (52%)	125 (53%)	0.8
- Truncating variants	8 (0.1)	20 (0.1)	28 (0.1)	7 (11%)	19 (11%)	26 (11%)	1.0
- Missense variants	44 (0.7)	101 (0.6)	145 (0.6)	31 (48%)	74 (43%)	105 (44%)	0.5
- Others^a^	6 (0.1)	13 (0.1)	19 (0.1)	6 (9%)	13 (8%)	19 (8%)	0.6
Most interesting variants	15 (0.2)	36 (0.2)	51 (0.2)	14 (22%)	30 (17%)	44 (19%)	0.5

^a^ Potential exonic splice variants; start-loss and stop-loss variants

**Table 4:** Most interesting variants. All variants were heterozygous. Clear pathogenic mutations are shown in bold

Patient ID	Sex	Age at last contact	*Gene*	Mutation (heterozygous)	Predicted gene phenotype	Phenotype of patient
A) Variants identified in addition to a known germline mutation (group K)						
Patient with two additional truncating mutations						
**49197**	**m**	**1**	***NF1*** *(NM_001042492.2)*	c.4107C > A;p.Tyr1369*	Neurofibromatosis type 1	LFS (*TP53* mutation): rhabdomyosarcoma and neuroblastoma (1 y)
			*WRN (NM_000553.4)*	c.4216C > T;p.Arg1406*	Werner syndrome	
Patients with one additional truncating mutation						
41393	f	23	*CHEK2 (NM_007194.3)*	c.1100delC;p.Thr376Metfs*15	susceptibility breast cancer	HDGC (*CDH1* mutation)
35145	f	48	*ERCC3 (NM_000122.1)*	c.1421dup;p.Asp474Glufs*2	susceptibility breast cancer (biallelic mutations: Xeroderma pigmentosum (B))	HNPCC (*MSH6* mutation)
38982	m	30	*FANCD2 (NM_033084.3)*	c.990-1G > A	cancer susceptibility? (biallelic mutations: Fanconi anaemia (D2))	MAP (MUTYH mutations)
34429	m	59	*PALLD (NM_001166109.1)*	c.2269C > T;p.Gln757*	susceptibility pancreatic cancer	HNPCC (*MSH2* mutation)
30560	m	26	*SDHA (NM_004168.3)*	c.1432_1432 + 1del;p.?	hereditary paraganglioma-pheochromocytoma syndrome	HNPCC (*MSH2* mutation)
32679	f	32	*XPC (NM_004628.4)*	c.622-2A > G	cancer susceptibility? (biallelic mutations: Xeroderma pigmentosum (C))	MAP (*MUTYH* mutations)
Patients with additional potential pathogenic missense / stoploss variants						
45126	f	53	*BRCA1 (NM_007300.3)*	c.5068G > T;p.Ala1690Ser	HBOC	HDGC (*CDH1* mutation); gastric and breast cancer, sister breast cancer
9691	f	59	*MLH1 (NM_000249.3)*	c.65G > C;p.Gly22Ala	HNPCC	JPS (*SMAD4* mutation)
47360	f	24	*MSH2 (NM_000251.2)*	c.1607A > G;p.Asn536Ser	HNPCC	JPS (*BMPR1A* mutation)
36206	m	57	*MSH6 (NM_000179.2)*	c.3600A > G;p.Ile1200Met	HNPCC	FAP (*APC* mutation), two siblings CRC (~ 55 y)
24320	f	68	*MSH6 (NM_000179.2)*	c.3664 T > G;p.Phe1222Val	HNPCC	FAP (*APC* mutation), CRC (68 y), sister CRC (< 64 y)
29267	f	65	*POLE (NM_006231.3)*	c.861 T > A;p.Asp287Glu	PPAP	PJS (*STK11* mutation)
44792	m	39	*RAD51B (NM_133509.3)*	c.1155A > T;p.*385Tyrext*64	breast cancer	HNPCC (*MSH2* mutation)
B) Newly identified variants in patients with previously unknown cause (group U)						
Patients with likely pathogenic mutations						
26356	f	9	***PMS2 (NM_000535.5)***	**c.1A > T;p.Met1?**	constitutional mismatch repair deficiency syndrome	multiple tumors: lymphoma (9 y), ALL, CRC (11 y), glioblastoma (15 y); two siblings died because of medulloblastoma / glioblastoma and ALL / astrocytoma
				c.2117delA;p.Lys706Serfs*19		
38569	m	58	***POLD1*** *(NM_002691.3)*	**c.1379 T > G;p.Lys460Arg**	PPAP	adenomatous polyposis (58 y, > 50 colorectal adenomas) + renal cancer (59 y)
			*PMS2 (NM_000535.5)*	c.2153 T > C;p.Leu718Pro	HNPCC	
40816	f	33	***PTEN*** *(NM_000314.4)*	**c.83 T > C;p.Ile28Thr**	Cowden syndrome	CRC (32 y), duodenal lymphangiectasis, liver hemangioma, polyps or cysts in the ovary and polyps in the cervix uteri; paternal grandmother: cancer of the cervix uteri, CRC, and gastric cancer (> 70 y)
Patients with two potential pathogenic variants in different genes (at least one truncating mutation)						
13225	m	67	*BARD1 (NM_000465.3)*	c.1690C > T;p.Gln564*	susceptibility breast cancer and neuroblastoma	adenomatous + hyperplastic polyps, brother CRC (40 y)
			*XRCC3 (NM_001100119.1)*	c.954_957del; p.Ser319Profs*49	susceptibility several cancer types	
44298	m	73	*FANCF (NM_022725.3)*	c.1087C > T;p.Gln363*	cancer susceptibility? (biallelic mutations: Fanconi anaemia (F))	duodenal polyposis (*n* = 15, only one was investigated histologically: adenoma), further polyps in small bowel, two colorectal adenomas (73 y)
			*BMPR1A (NM_004329.2)*	c.712C > G;p.Arg238Gly	juvenile polyposis syndrome	
38124	m	62	*MLH3 (NM_001040108.1)*	c.1798C > T;p.Arg600*	HNPCC (?)	member of a family with suspected hyperplastic polyposis syndrome in one cousin, several other family members have some colorectal polyps or colorectal cancer. For patient 38124 only some polyps are reported, no histologic report is available. Five other affected family members have been also investigated with the gene panel, but no one else carries the variants in MLH3 or MUTYH
			*MUTYH (NM_001128425.1)*	c.667A > G;p.Ile223Val	*MUTYH*-associated polyposis (biallelic mutations)	
35847	f	64	*POLE (NM_006231.3)*	c.6623del;p.Gln2208Argfs*4	PPAP	adenomatous polyps + familial CRC + other tumors
			*APC (NM_000038.5)*	c.5009C > T;p.Ala1670Val	FAP	
Patients with truncating mutations						
15263	m	71	*ATM (NM_000051.3)*	c.5932G > T;p.Glu1978*	susceptibility breast cancer (biallelic mutations: Ataxia-Telangiectasia)	hyperplastic polyposis (father of ID 48355)
48355	f	55	*ATM (NM_000051.3)*	c.5932G > T;p.Glu1978*	susceptibility breast cancer (biallelic mutations: Ataxia-Telangiectasia)	hyperplastic polyposis (daughter of ID 15263)
45647	f	59	*BRIP1 (NM_032043.2)*	c.2684_2687delCCAT;p.Ser895*	susceptibility breast and ovarian cancer (biallelic mutations: Fanconi anaemia (J))	hyperplastic polyposis, father pancreatic cancer
46448	f	28	*CHEK2 (NM_007194.3)*	c.444 + 1G > A	susceptibility breast cancer	medullary thyroid cancer (27 y)
44401	f	73	*CHEK2 (NM_007194.3)*	c.1100delC;p.Thr376Metfs*15	susceptibility breast cancer	follicular thyroid cancer (63 y), breast cancer (70 y), paraganglioma (72 y), renal angiomyolipoma
48835	m	32	*CHEK2 (NM_007194.3)*	c.1100delC;p.Thr376Metfs*15	susceptibility breast cancer	papilly thyroid cancer, NHL (both 31 y), in family thyroid, prostatic and breast cancer
32119	f	64	*CHEK2 (NM_007194.3)*	c.1100delC;p.Thr376Metfs*15	susceptibility breast cancer	thyroid cancer, breast cancer (55 y)
22715	f	67	*CHEK2 (NM_007194.3)*	c.1555C > T;p.Arg519*	susceptibility breast cancer	uterine cancer (32 y), several adenomas, two with high grade dysplasia (62 + 71 y), son with colorectal polyps
47863	f	61	*EXT1 (NM_000127.2)*	c.1483C > T;p.Gln495*	multiple osteochondromas / exostoses	several tumors in family
10317	m	35	*FANCE (NM_021922.2)*	c.248 + 2dupT	biallelic mutations: Fanconi anaemia	sarcoma (35 y); in family: neuroblastoma, testicular cancer, bronchial cancer
19347	f	61	*FANCL (NM_001114636.1)*	c.1111_1114dupATTA; p.Thr372Asnfs*13	biallelic mutations: Fanconi anaemia	bilateral breast cancer (43 + 53 y), bronchial cancer (61 y); in family: sarcoma, uterine cancer, pancreatic cancer, bronchial cancer
47257	f	56	*RAD51C (NM_058216.2)*	c.706-1G > A	hereditary breast and ovarian cancer	two synchronous CRCs, 10 colorectal adenomas; endometrial cancer in family
28219	m	48	*SDHA (NM_004168.3)*	c.995dup;p.Val333Cysfs*8	hereditary paraganglioma-pheochromocytoma syndrome	polyposis
10083	m	33	*XPC (NM_004628.4)*	c.780-1G > A	biallelic mutations: Xeroderma pigmentosum (C)	suspected LFS
Patients with potential pathogenic missense variants						
16335	m	40	*APC (NM_000038.5)*	c.1631 T > C;p.Ile544Thr	FAP	suspected LFS
37416	m	29	*APC (NM_000038.5)*	c.4292 T > A;p.Met1431Lys	FAP	early CRC (before 30y), MSS in tumor tissue
37982	m	38	*APC (NM_000038.5)*	c.281G > A;p.Arg94His	FAP	CRC (before 40y), MSS in tumor tissue
44716	m	52	*APC (NM_000038.5)*	c.7645C > T;p.Arg2549Cys	FAP	Gastrointestinal stromal tumor
17052	f	46	*BMPR1A (NM_004329.2)*	c.1022G > A;p.Gly341Asp	juvenile polyposis syndrome	ovarian cancer (44 y), CRC and breast cancer in family history
46581	f	33	*BMPR1A (NM_004329.2)*	c.1243G > A;p.Glu415Lys	juvenile polyposis syndrome	bladder cancer 32y
18225	f	12	*CDH1 (NM_004360.3)*	c.1298A > G;p.Asp433Gly	HDGC	suspected LFS
45312	f	33	*MSH6 (NM_000179.2)*	c.3724C > T;p.Arg1242Cys	HNPCC	diffuse gastric cancer (33 y), no HNPCC typical findings in tumor tissue, no *CDH1* mutation, suspicious family history
43219	f	65	*POLD1 (NM_002691.3)*	c.961G > A;p.Gly321Ser	PPAP	medullary thyroid cancer (64 y), in family: pancreatic and breast cancer

*ALL* = Acute Lymphoblastic Leukemia, *CRC* = Colorectal Cancer, *FAP* = Familial adenomatous polyposis, *HBOC* = hereditary breast and ovarian cancer, *HDGC* = hereditary diffuse gastric cancer, *HNPCC* = hereditary nonpolyposis colorectal cancer, *JPS* = Juvenile Polyposis Syndrome, *LFS* = *Li*-Fraumeni Syndrome, *MAP* = *MUTYH*-associated Polyposis, *MSS* = microsatellite stability, *PJS* = Peutz-Jeghers Syndrome, *PPAP* = Polymerase-Proofreading Associated Polyposis, *y* = years of age, age at first diagnosis, * = termination codon

Of the 148 cancer panel genes, the number of additional germline variants detected was: zero in 62 genes; 1–2 in 58 genes; and 3–12 in 28 genes (Additional file 3: Table S2, Additional file 6: Table S5, and Additional file 2: Figure S3). No significant inter-group difference was observed in the proportion of patients with variants in the most frequently mutated, moderately penetrant genes *CHEK2* and *ATM* (group U: 18 variants in one patient each, 10%; group K: 3 variants in 2 patients, 3%; *p* = 0.1). For the other frequently mutated genes (≥ 3 variants), a inter-group difference was observed for the distribution of variants. Here, variants in seven genes were detected in group U only (*APC, BMPR1A, FANCI, GALNT12, PMS2, POLD1, PTCH1);* whereas variants in seven other genes were slightly overrepresented in group K (*BUB1B, DICER1, EXO1, MSH6, MTOR, PALB2, POLE*). For nearly half of the genes, the percentage of variants in genes with ≥3 variants was comparable in both groups (Additional file 6: Table S5 and Additional file 2: Figure S3).

To identify further potential causative mutations among the 192 additional germline variants in both patient groups, all truncating mutations as well as start-loss and stop-loss variants were selected (*n* = 30). Of the predicted pathogenic missense variants, those were selected (*n* = 21), which were located in the most common high penetrance genes for HTS (*APC, BMPR1A, BRCA1, CDH1, MLH1, MSH2, MSH6, MUTYH, NF1, PMS2, POLD1 (polymerase domain), POLE (polymerase domain), PTEN*; the 192 variants did not include variants in *BRCA2, SMAD4, STK11,* or *TP53*). An overview of the 51 most interesting variants is provided in Table 4, all of them were heterozygous. All 51 variants were confirmed by Sanger sequencing (except in three cases, for whom no further DNA was available).

In both groups, the proportion of the most interesting variants per patient was 0.2 (15 variants in 14 patients of group K (22%) and 36 variants in 30 patients of group U (17%); *p* = 0.5) (Table 3). Interesting findings in group K and U are described below.

### Patients with a known causative germline mutation (group K)

The most interesting finding was an *NF1* nonsense mutation (c.4107C > A;p.Tyr1369*) in a male proband with a known *TP53* frameshift mutation*.* He was diagnosed with a neuroblastoma and a rhabdomyosarcoma at the age of one year, and died at the age of two years. In addition, he carried a heterozygous nonsense mutation in *WRN.* No information was available concerning whether or not the patient exhibited cutaneous symptoms of any form of neurofibromatosis or specific symptoms of Werner syndrome.

In four patients with *APC*-related familial adenomatous polyposis or *SMAD4/BMPR1A*-related juvenile polyposis syndrome, an additional potential pathogenic missense variant in an MMR gene (*MLH1, MSH2, MSH6*) was detected.

### Patients with a previously unexplained suspected HTS (group U)

The most interesting findings included two compound heterozygous mutations in *PMS2* in one patient (one start-loss mutation: c.1A > T;p.Met1? and one frameshift mutation: c.2117delA;p.Lys706Serfs*19). The phenotype of the patient was compatible with a constitutional mismatch repair deficiency syndrome (CMMRD) (Table 4B, ID 26356).

Furthermore, a *POLD1* missense variant in the proofreading domain of the protein (c.1379 T > G;p.Leu460Arg) was identified in a patient with colorectal adenomatous polyposis and papillary renal cancer (Table 4B, ID 38569). The alteration p.Leu460Arg affects a strongly conserved leucine in the exonuclease (proofreading) domain of POLD1 (Additional file 2: Figure S4). The patient also carried a *PMS2* missense variant. However, immunohistochemical analysis of the renal cancer tissue revealed no loss of PMS2 expression (or of the expression of MLH1, MSH2, and MSH6), and the tumor exhibited microsatellite stability.

The *PTEN* missense variant c.83 T > C;p.Ile28Thr was detected in one patient with early onset CRC (Table 4B, ID 40816). This variant affects a highly conserved nucleotide and amino acid in the region of a functionally important phosphatase domain of the PTEN protein. The variant has not been described previously in either patients with suspected Cowden syndrome or controls. However, pathogenic missense variants in flanking codons (e. g., codon 27 and 30) have been reported in Cowden syndrome patients [11–13].

In two more patients, possibly pathogenic variants were detected in *BMPR1A* and *MSH6*, respectively. Both were compatible with the phenotype (Table 4B, IDs 44298 and 45312).

Four of the five patients from group U with a truncating *CHEK2* mutation had thyroid cancer (clinical details provided in Table 4B).

### Incidental findings in both patient groups

Incidental (secondary) findings with presumptive predictive value were generated in both groups. This included the identification of two frameshift mutations in *SDHA*, one nonsense mutation in *EXT1,* one potential splice site mutation in *RAD51C,* and a *POLD1* missense variant within the polymerase domain (c.961G > A;p.Gly321Ser). The available clinical reports contained no evidence for a personal or family history of hereditary paraganglioma-pheochromocytoma syndrome; multiple osteochondromas / exostoses; hereditary breast and ovarian cancer (HBOC); or Polymerase proofreading-associated polyposis (PPAP).

A potential heterozygous carrier status for an autosomal recessive HTS was found in 30% (70 / 237) of the total cohort (group K: 41% (26 / 64); group U: 25% (44 / 173)). This was based on the mode of inheritance listed in Additional file 3: Table S2.

In total, seven patients (3%) carried two variants in the same gene (Additional file 7: Table S6). Four of these genes are known to cause recessive conditions (*ERCC2* for Xeroderma pigmentosum; and MSH2, MSH6, or PMS2 for CMMRD). However, with the exception of the *PMS2*-related CMMRD, no patient displayed clinical signs of the respective recessive condition. In three of the four patients from group K, the additional germline variant (appearing in the list of 192 variants) was in the same gene than the known heterozygous pathogenic germline mutation (*MSH2* and *MSH6*, respectively), as already known using Sanger sequencing during routine diagnostics. In two of the *MSH2* and *MSH6* mutation carriers, segregation analysis was possible. This demonstrated that both were located on the same allele.

## Discussion

In a proportion of suspected HTS patients, no germline mutation is identified in the most likely affected genes. NGS-based multi-gene analysis is a powerful approach to the detection of mutations in genes that are not primarily suspected on the basis of clinical criteria, or in HTS with several causative genes.

To determine the diagnostic yield and clinical utility of a comprehensive gene panel of 148 HTS genes, targeted sequencing was performed in 173 patients with suspected but unexplained HTS (group U) and 64 HTS patients with known pathogenic germline mutations in established HTS genes (group K).

All but one of the previously known germline mutations, benign variants, or VUS (*n* = 260) were identified, indicating a sensitivity of 99.6%. This demonstrates that the multi-gene panel approach identified a broad spectrum of variants, ranging from single base pair substitutions to larger deletions/duplications (14–65 bp). In genes screened prior to the study during routine diagnostics by Sanger sequencing, no additional germline variant was identified by NGS.

After stringent filtering, 192 rare loss-of-function mutations and potential pathogenic missense variants remained. Interestingly, the percentage of additional germline variants was similar in both study groups. In addition, no significant inter-group difference was observed for variants in the most frequently mutated genes *CHEK2* and *ATM* (*p* = 0.1). The identification of these variants in patients with genetically explained disease (group K) highlights the fact that caution must be exercised when interpreting their role in patients with unexplained disease, since they cannot explain the respective disease alone [14]. Moreover, the effect size of moderately penetrant risk alleles is strongly dependent on individual and family history.

Furthermore, no significant inter-group difference was found for mutation type or the presence of the 51 most interesting variants (Table 3). In group K and group U respectively, 22 and 17% of probands, harbored (additional) rare potential causative variants (corresponding to the most interesting variants in Table 4).

In one patient from group K (2%), a second clearly pathogenic, high penetrant germline mutation in *NF1* was identified in addition to a known frameshift mutation in *TP53*. Due to the combination of tumors in this individual (neuroblastoma and rhabdomyosarcoma), Li-Fraumeni syndrome was suspected. However, both tumors also occur in children with neurofibromatosis type 1 [15]. A combination of pathogenic mutations would be overlooked in a stepwise approach limited to the most likely affected genes. In such patients, an atypical clinical presentation or an unusually broad spectrum of tumors might prompt the analysis of further genes. However, in routine practice, clinical information is often limited, or the patient is too young to have yet presented with the typical spectrum of signs and symptoms, thus leading to delayed diagnosis.

The present analyses also identified five patients in group U (3%) with two potential pathogenic variants in different genes. This is consistent with the results of previous studies, which have reported rates of between 0.1 and 3% [7, 16–18] or up to 7.5%, when potentially pathogenic missense mutations were included [19]. These cases may involve digenic inheritance, co-inheritance of genetic modifiers, or clear pathogenic mutations [20, 21]. The presence of 2 or more inherited cancer predisposition alleles in the same individual was also described by Whitworth et al. [22] and was called multilocus inherited neoplasia alleles syndrome (MINAS).

In three patients (2%) from group U, the genetic cause was identified to a high degree of certainty. The phenotype of the patient with a compound-heterozygous *PMS2* germline mutation (the patient was previously described in Adam et al. [23]), was suggestive of CMMRD at the age of 15 years, however, on initial referral at the age of 9 years, the most likely differential diagnosis had been Li-Fraumeni syndrome. If multi-gene analysis had been available at that time, a CMMRD diagnosis could have been assigned much earlier and specific surveillance measures and predictive testing offered.

In a patient who showed features compatible with Cowden syndrome, a probable pathogenic *PTEN* missense variant was identified. Lynch syndrome had been considered the most likely clinical diagnosis at first due to the presentation of an early onset CRC and the absence of the major cancer types of Cowden syndrome, again pointing to the broad phenotypic overlap between well known tumor syndromes.

The *POLD1* mutation c.1379 T > G;p.Leu460Arg in a patient with colorectal adenomatous polyposis and renal cancer is located in the vicinity of the known hotspot mutation c.1433G > A;p.Ser478Asn. Specific germline mutations in the proofreading domain of *POLD1* have recently been identified as underlying rare cause of multiple colorectal adenomas and carcinomas [24]. This condition is termed Polymerase proofreading-associated polyposis (PPAP) [25]. The identified missense variant is likely to be pathogenic, since it introduces a charged side chain into the hydrophobic pocket within the exonuclease domain and thereby distorts the proteins’ structure (Additional file 2: Figure S4). Very rarely affected causative genes for adenomatous polyposis such as *POLD1, POLE, NTHL1,* or *MSH3*, might collectively clarify a sufficient number of cases. However, since these polyposis types do not present with a specific phenotype, a genetic diagnosis is usually only possible using a multi-gene panel approach.

Interestingly, in the present study, rare *APC* missense variants were detected in group U only (Additional file 6: Table S5). These clustered in patients with familial / early onset CRC (3/38 = 8% compared to the remaining patients of group U (2/135 = 1.5%)), however, the difference was not statistically significant (*p* = 0.07). Two of the variants in the present CRC patients were located in the β-catenin down-regulating domain (c.4292 T > A;p.Met1431Lys and c.5009C > T;p.Ala1670Val). Rare germline *APC* missense mutations are not part of the typical mutation spectrum of *APC*-related familial adenomatous polyposis (FAP). However, research suggests that they are significantly overrepresented in patients with colorectal adenomas, particularly variants in the functionally important β-catenin down-regulating domain [26]. Notably, in two other gene panel studies, an *APC* missense variant was detected in 4% (44/1137) of patients with suspected, but unexplained, Lynch syndrome and in 5% (53/1058) of unselected CRC patients [27, 28]. However, even when the results of all three studies are taken together, *APC* missense variants are not enriched in CRC cases (4.5%) compared to individuals from the general population (8.9%) (based on the gnomAD-database where 12,267 alleles with rare (MAF ≤ 1%) *APC* missense variants were detected in exome and genome data of 138,632 individuals). Nonetheless, the potential relevance of specific *APC* missense mutations cannot be excluded.

Four of the five patients from group U with a truncating *CHEK2* mutation had thyroid cancer (in group K only one patient carried a truncating *CHEK2* mutation, no thyroid cancer is reported). Interestingly, a previous study with 468 unselected patients with papillary thyroid cancer found that carriers of truncating *CHEK2* mutations had a six-fold increase in risk for a papillary thyroid carcinoma [29]. Thus, the tumor spectrum in patients with pathogenic *CHEK2* variants seems to include thyroid cancer and appears to be broader than previously thought. However, additional larger studies are warranted to evaluate tumor risk in these patients.

Since most of the other patients with one of the most interesting variants did not show the classical phenotype, these variants might represent moderately penetrant risk alleles, or modifiers which act in conjunction with other risk factors, rather than highly penetrant mutations.

Furthermore, these variants might represent incidental (secondary) findings with presumptive predictive value. Comprehensive genetic approaches such as exome studies are prone to generate predictive information. Since the present gene panel included a limited number of causal genes for a circumscribed phenotype (HTS), the overall likelihood of predictive information was expected to be low. However, the differentiation between diagnostic and predictive information might be problematic in some cases. A predictive result was assumed if a pathogenic mutation was found and the known clinical spectrum of the altered gene did not overlap with the respective phenotype. Nonetheless, the possibility that predictive results point to a broader tumor spectrum cannot be excluded. In line with this, predictive genetic information was generated for both study groups, e. g., the identification of truncating mutations in *SDHA* and *EXT1*. Disclosure of predictive information may be challenging, and thus pre- and posttest counseling may be required, in particular since penetrance in this context may be reduced and thus the appropriate surveillance strategy is unclear. Particular caution is required in such cases with regard to prophylactic surgery [30]. To reduce the number of secondary findings, a panel approach (virtual panel) can be used to restrict the analyses to genes that are compatible with the phenotype of interest.

Based on the applied variant screening and filter approach, some potentially causative rare variants might have been missed in this study. In particular, large genomic rearrangements, and synonymous variants except for the first or last three exonic bases, were not considered. Since those variants might explain some more cases, the diagnostic yield might be even higher, if all possibly causative variants are included. However, the used filter and analysis approach is very similar to the current procedure in routine genetic panel diagnostics.

Previous studies have investigated the diagnostic yield of gene panels for several HTS, in particular HBOC and hereditary CRC (an (incomplete) overview of these studies is provided in Additional file 8: Table S7). The results of gene panel studies are heavily dependent on the study inclusion criteria; the number of patients (varying between 20 and 252,223); the number of previously screened genes (mostly *BRCA1/2* or investigation of MMR); the number of included genes (range of 7–112); and the classification of variants. The results of these studies are therefore difficult to compare. Overall, in these studies pathogenic mutations were identified in 2–33% of patients with suspected HTS and in 2–18% of unaffected controls [18, 19]. However, in around half of these studies, only index patients without previous Sanger sequencing of the most relevant genes were investigated, resulting in a relatively high proportion of solved cases. In virtually all previous studies, no adequate cohorts of patients with a known pathogenic mutation were included to demonstrate the impact of additional rare variants in genetically explained cases. One exception was the study of Kurian et al. [31], which investigated 57 women with known BRCA1/2 mutations. However, only one patient (2%) carried a monoallelic *MUTYH* mutation.

## Conclusions

In conclusion, the present data highlight the importance of analyzing all genes compatible with the phenotype of interest. The study demonstrated that the application of a comprehensive gene panel is an efficient approach to the identification of causative mutations, particularly in patients with atypical or mild phenotypes or very heterogeneous monogenic conditions. However, the diagnostic yield is limited in patients in whom a prior investigation of likely HTS genes has been conducted. The major strength of the present study was the comparison of genetically unexplained patients with a substantial group of patients with previously confirmed HTS. Moderately penetrant risk alleles occur in both patient groups and are likely to act as modifiers. The present findings also show that some patients harbor (likely) pathogenic mutations in more than one established HTS gene. This renders the interpretation of the phenotypic contribution of those alterations challenging, and indicates that the classification of a variant as pathogenic must be performed with caution. Furthermore, the potential of gene panels for the generation of incidental predictive information must be considered. In addition to the facilitation of diagnostics, extended analysis via comprehensive gene panels will broaden knowledge concerning the tumor spectra of established HTS genes.

## DATABASES / URLs

Cancer Gene Census, Sanger Institute: http://cancer.sanger.ac.uk/cosmic/census/.

dbSNP (The Single Nucleotide Polymorphism database): www.ncbi.nlm.nih.gov/SNP/.

Ensembl Genome Browser: https://www.ensembl.org

EVS (Exome Variant Server): http://evs.gs.washington.edu/EVS/.

Genome Aggregation Database (gnomAD): http://gnomad.broadinstitute.org

HGMD (Human Gene Mutation Database): http://hgmd.org

IGV (Integrative Genomics Viewer): www.broadinstitute.org/igv/.

Locus-specific mutation databases: www.lovd.nl/.

MutationTaster: www.mutationtaster.org

NCBI: www.ncbi.nlm.nih.gov/.

NNSPLICE 0.9: http://www.fruitfly.org/seq_tools/splice.html

PolyPhen-2: http://genetics.bwh.harvard.edu/pph2/.

Primer3: http://bioinfo.ut.ee/primer3/

SIFT (Sorting Intolerant From Tolerant): http://sift.jcvi.org.

TGP (1000 Genomes Project): www.1000genomes.org

UCSC Genome Browser: http://genome.ucsc.edu

## Additional files

Additional file 1: **Table S1.** List of all 64 patients with known germline mutations = group K. With the exception of mutations in *MUTYH*, all mutations were in a heterozygous state. (XLSX 16 kb)

Additional file 2: **Figure S1.** Number of genes investigated previously within the context of routine diagnostics and inconspicuous tumor tissue findings in cases of suspected Lynch syndrome in patients with (blue) or without (red) a known germline mutation prior to the present gene panel investigation. **Figure S2.** Percentage of patients with known mutations (blue) and patients without known mutations (red) carrying 0–4 additional variants. **Figure S3.** Percentage of patients with variants in the most frequently mutated 28 genes (≥ 3 variants per gene). **Figure S4. Top:** Sequence logo presentation of protein sequence conservation of POLD1, comprising Leu460 and residues of relevance to its structural role. Highly conserved, hydrophobic residues significantly contributing to the hydrophobic pocket are indicated by yellow shading (mainly Leu469, Tyr472, Leu474, and Tyr396). **Bottom:** Overview of the structure of POLD1 and its domain architecture (left) (DOCX 527 kb)

Additional file 3: **Table S2.** Overview of all 148 investigated genes, including number of identified variants in the present study and details of sequencing quality (XLSX 32 kb)

Additional file 4: **Table S3.** List of detected deletions/duplications with a size of 14–65 bp and two indels encompassing five to 11 bp (XLSX 11 kb)

Additional file 5: **Table S4.** List of the 192 additional variants that matched the filter criteria (variants in addition to a known germline mutation (group K) (A) or newly identified variants in patients with unknown cause (group U) (B)) (XLSX 30 kb)

Additional file 6: **Table S5.** List of the 28 genes with more than 3 variants per gene (XLSX 13 kb)

Additional file 7: **Table S6.** List of patients with two variants in the same gene (based on the list of 192 variants and known mutations) (XLSX 19 kb)

Additional file 8: **Table S7.** Literature review. (DOCX 54 kb)
