# Supplementary material for: Diagnostic yield and clinical utility of a comprehensive gene panel for hereditary tumor syndromes
Source: Hered Cancer Clin Pract. 2019 Jan 23;17:5. doi: 10.1186/s13053-018-0102-4 (PMC6343270; doi:10.1186/s13053-018-0102-4)
Supplement: Supplementary file 2 — Figure S1. Number of genes investigated previously within the context of routine diagnostics and inconspicuous tumor tissue findings in cases of suspected Lynch syndrome in patients with (blue) or without (red) a known germline mutation prior to the present gene panel investigation. Figure S2. Percentage of patients with known mutations (blue) and patients without known mutations (red) carrying 0–4 additional variants. Figure S3. Percentage of patients with variants in the most frequently mutated 28 genes (≥ 3 variants per gene). Figure S4. Top: Sequence logo presentation of protein sequence conservation of POLD1, comprising Leu460 and residues of relevance to its structural role. Highly conserved, hydrophobic residues significantly contributing to the hydrophobic pocket are indicated by yellow shading (mainly Leu469, Tyr472, Leu474, and Tyr396). Bottom: Overview of the structure of POLD1 and its domain architecture (left) (DOCX 527 kb) [file 13053_2018_102_MOESM2_ESM.docx]

**Online supporting information**

**Henn et al.**

**Diagnostic yield of a comprehensive gene panel**

**for hereditary tumor syndromes**

**Online methods**

**Patients / data collection**

All 237 index patients had been referred to the Institute of Human Genetics in Bonn from within Germany for molecular genetic investigation of a suspected HTS. 218 patients (92%) were unrelated index patients, the remaining 19 cases were relatives (4 healthy parents and 15 affected relatives) from a total of 13 families without known pathogenic germline mutation.

The cohort of the present study comprised on the one hand 64 HTS index patients with a confirmed pathogenic germline mutation in an HTS gene (group K, for known mutation). These cases presented with a broad spectrum of 14 distinct HTS, including Lynch syndrome, colorectal polyposis, Li-Fraumeni syndrome, Cowden syndrome, and hereditary diffuse gastric cancer (Table 1A), caused by 54 germline single base pair substitutions or insertions / deletions of 17 genes and ten large deletions comprising two to eight exons (Suppl. Table S1).

On the other hand 173 patients with a suspected, but genetically unexplained, HTS were included (group U, for previously unknown cause). All cases presented with an early age at onset, multiple primary tumors, and / or a striking familial clustering of various tumors and were grouped into six different phenotypic classes: suspected hereditary colorectal cancer (CRC); colorectal polyposis; Li-Fraumeni syndrome; Cowden syndrome; hereditary gastric cancer; and others (Table 1B). However, no mutation in the most likely affected genes had been identified during routine diagnostics.

Prior to the present study and within the context of routine diagnostics, leukocyte DNA from almost all 237 patients was screened for germline mutations in the gene responsible for the suspected HTS and, if applicable, the most likely differential diagnoses. This involved the use of standard procedures, such as Sanger sequencing of the coding regions and deletion/duplication analysis by Multiplex ligation-dependent probe amplification (MLPA), as described elsewhere for patients with adenomatous polyposis syndromes ([1](#_ENREF_1)). In patients with suspected hereditary non-polyposis colorectal cancer (HNPCC / Lynch syndrome), microsatellite analysis and/or immunohisto-chemical analysis of the tumor tissue for mismatch repair (MMR) proteins was performed ([2](#_ENREF_2)).

In group K, at least one gene had been investigated in 100% of patients and 2-5 genes in 28% of patients in order to identify the causal germline mutation (Suppl. Fig. S1). In 91% of index patients from group U, at least one gene or tumor tissue (in the case of suspected Lynch syndrome) had been investigated during routine diagnostics. In 19 % of index patients from group U, a total of 2-5 genes had been analyzed. These patients encompassed all six phenotype groups. In 9% of individuals from group U, no genes had been investigated previously, since affected relatives had undergone genetic investigation (n=8) or no appropriate genetic diagnostics were available at the time of referral (n=7).

**Table S1.** List of all 64 patients with known germline mutations = group K. With the exception of mutations in *MUTYH*, all mutations were in a heterozygous state.

**Table S2.** Overview of all 148 investigated genes, including number of identified variants in the present study and details of sequencing quality

**Table S3.** List of detected deletions/duplications with a size of 14-65 bp and two indels encompassing five to 11 bp

**Table S4.** List of the 192 additional variants that matched the filter criteria (variants in addition to a known germline mutation (group K) (A) or newly identified variants in patients with unknown cause (group U) (B))

**Table S5.** List of the 28 genes with more than 3 variants per gene

**Table S6.** List of patients with two variants in the same gene (based on the list of 192 variants and known mutations)

**Table S7** Literature review

**
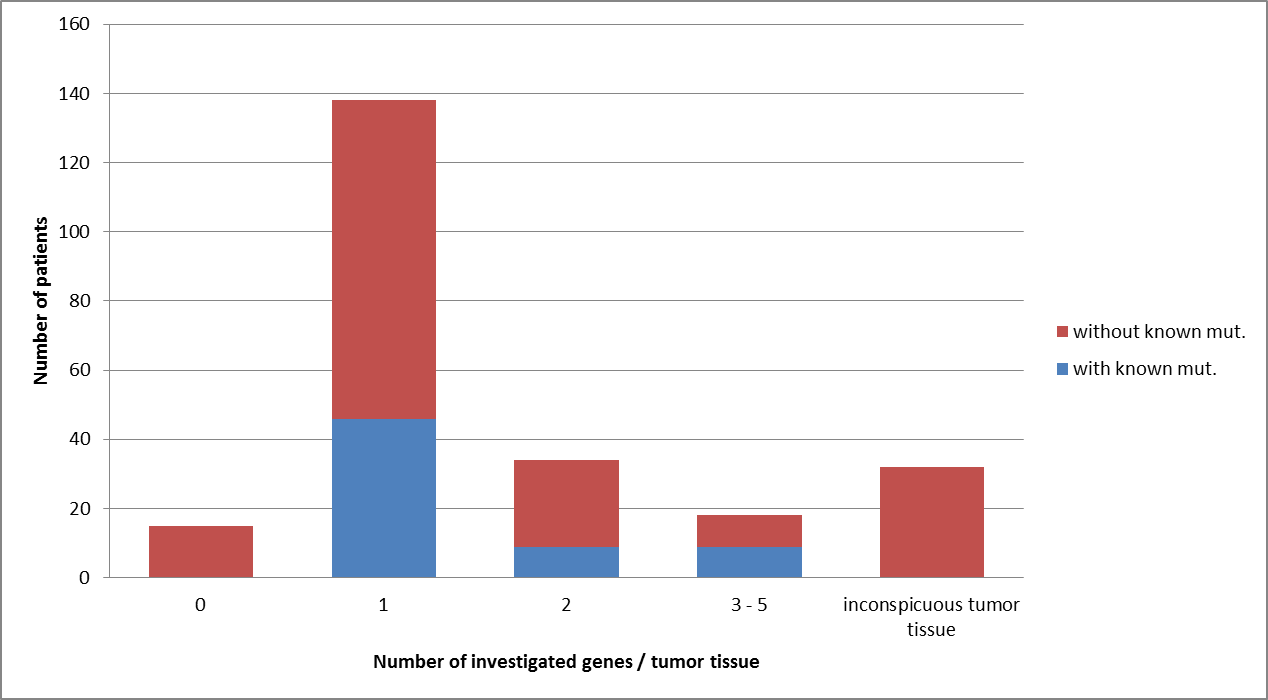
**

**Figure S1.** Number of genes investigated previously within the context of routine diagnostics and inconspicuous tumor tissue findings in cases of suspected Lynch syndrome in patients with (blue) or without (red) a known germline mutation prior to the present gene panel investigation. In 15 individuals, no genes had been investigated previously, since affected relatives had undergone genetic investigation (n=8) or no appropriate genetic diagnostics were available at the time of referral (n=7).

**
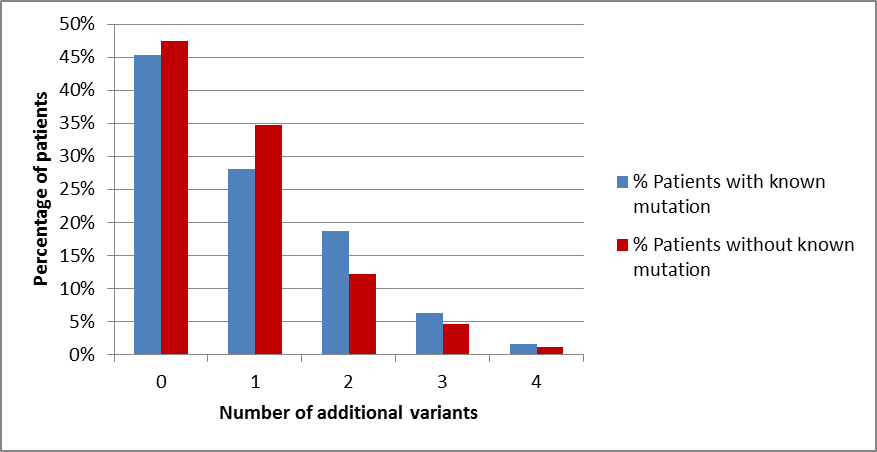
**

**Figure S2.** Percentage of patients with known mutations (blue) and patients without known mutations (red) carrying 0-4 additional variants

**
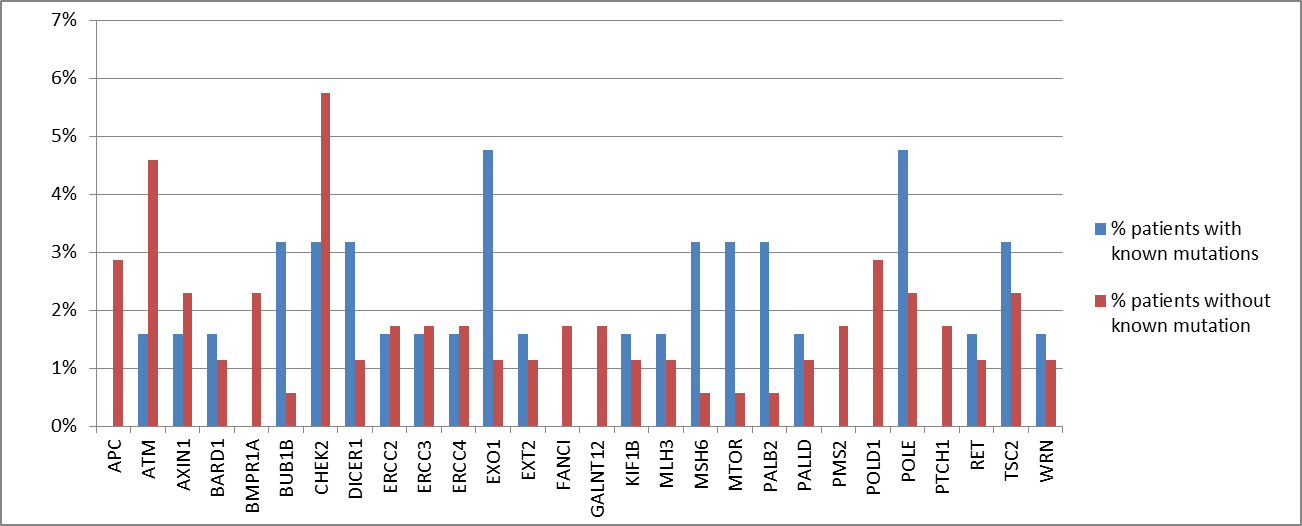
**

**Percentage of patients**

**Figure S3.** Percentage of patients with variants in the most frequently mutated 28 genes (≥ 3 variants per gene)


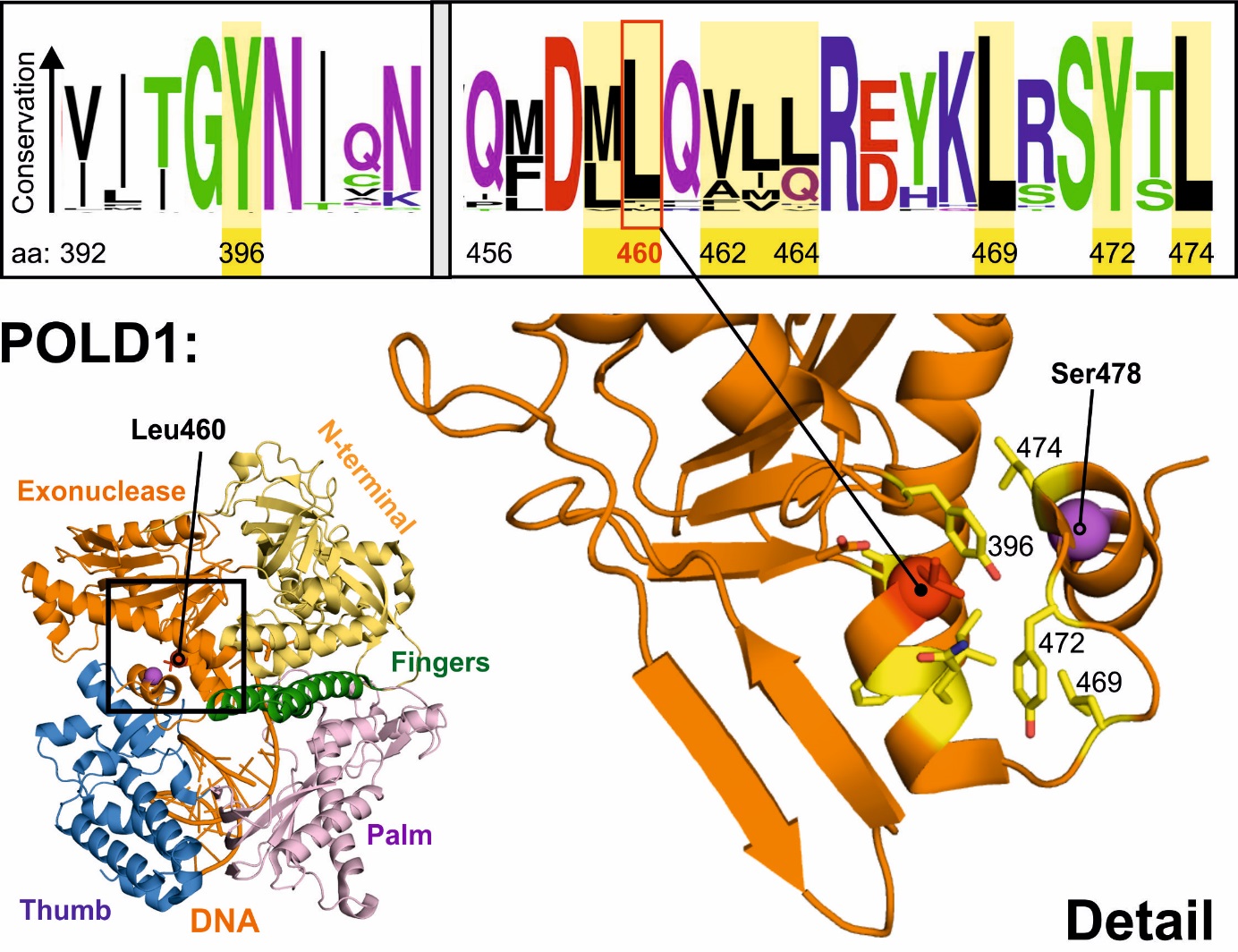


**Figure S4.** **Top:** Sequence logo presentation of protein sequence conservation of POLD1, comprising Leu460 and residues of relevance to its structural role. Highly conserved, hydrophobic residues significantly contributing to the hydrophobic pocket are indicated by yellow shading (mainly Leu469, Tyr472, Leu474, and Tyr396). **Bottom:** Overview of the structure of POLD1 and its domain architecture (left). The location of Leu460 within the exonuclease domain is indicated. The boxed area is shown in detail (right). One side of this hydrophobic pocket is formed by an alpha helix containing Ser478 (purple). Substitution of Leu460 (red) by the positively charged Arg residue will distort the local conformation by disrupting the hydrophobic pocket in which the residue is normally buried. The Leu460Arg substitution can therefore be expected to have a similar effect on protein function as the Ser478Asn mutation. The other conserved residues contributing to the hydrophobic pocket are indicated by yellow sticks. All position numbers correspond to the human POLD1 protein.

**Methods:** Nonredundant, full-length sequences of the POLD1 protein from diverse organisms were retrieved from the NCBI RefSeq protein database and aligned using T-Coffee ([3](#_ENREF_3)). The alignments were used to create Weblogo diagrams of local sequence conservation ([4](#_ENREF_4)). For structural analysis, the crystal structure of the highly homologous S. cerevisiae POLD1 protein (PDB 3IAY) was used. This shows a 51% identity overlap with the corresponding human protein within the crystallized sequence ([5](#_ENREF_5)). Structural figures were created using PyMOL v.1.4.1 (Schrödinger LLC).

**References**

1. Aretz S, Stienen D, Uhlhaas S, Pagenstecher C, Mangold E, Caspari R, et al. Large submicroscopic genomic APC deletions are a common cause of typical familial adenomatous polyposis. J Med Genet. 2005;42(2):185-92.

2. Steinke V, Holzapfel S, Loeffler M, Holinski-Feder E, Morak M, Schackert HK, et al. Evaluating the performance of clinical criteria for predicting mismatch repair gene mutations in Lynch syndrome: a comprehensive analysis of 3,671 families. Int J Cancer. 2014;135(1):69-77.

3. Notredame C, Higgins DG, Heringa J. T-Coffee: A novel method for fast and accurate multiple sequence alignment. J Mol Biol. 2000;302(1):205-17.

4. Crooks GE, Hon G, Chandonia JM, Brenner SE. WebLogo: a sequence logo generator. Genome Res. 2004;14(6):1188-90.

5. Swan MK, Johnson RE, Prakash L, Prakash S, Aggarwal AK. Structural basis of high-fidelity DNA synthesis by yeast DNA polymerase delta. Nat Struct Mol Biol. 2009;16(9):979-86.
