# Supplementary material for: Diagnostic yield and clinical utility of a comprehensive gene panel for hereditary tumor syndromes
Source: Hered Cancer Clin Pract. 2019 Jan 23;17:5. doi: 10.1186/s13053-018-0102-4 (PMC6343270; doi:10.1186/s13053-018-0102-4)
Supplement: Supplementary file 8 — Table S7. Literature review. (DOCX 54 kb) [file 13053_2018_102_MOESM8_ESM.docx]

**Table S7** Literature review.

| **Phenotype** | **Prescreening?** | **Number of investigated genes** | **Number of patients with previously unknown cause** | **Detection rate potential pathogenic mutations** | **VUS** | | **Reference** |
| --- | --- | --- | --- | --- | --- | --- | --- |
|  |  |  |  |  | **Percentage of patients with at least one VUS** | **Number of VUS** |  |
| **CRC, others** | **yes** | **148** | **173** | **17%, including 2% (likely) pathogenic** | **51%** | **130** | **Our study** |
| CRC | no | 9 | 626 | 14% | 10% | 64 | ([1](#_ENREF_1)) |
| CRC | deficient MMR CRCs without germline mutation in MMR gene (n = 129); proficient MMR CRCs (n = 221) | 36 | 1324: | 8% (truncating), 23% (potential pathogenic missense) | - | 584 VUS / 658 missense variants (89%) | ([2](#_ENREF_2)) |
|  |  |  | - 1231 CRC cases | 8% (truncating), 23% (potential pathogenic missense) |  |  |  |
|  |  |  | - 93 unaffected controls | 5% (truncating), 18% (potential pathogenic missense) |  |  |  |
| CRC | MMR genes | 112 | 274 | 6% | 7% | 19 | ([3](#_ENREF_3)) |
| CRC | no | 18 | 152 | 2% | 8% | 12 | ([4](#_ENREF_4)) |
| CRC | no | 7 | 31 | 19% | 10% | 3 | ([5](#_ENREF_5)) |
| CRC | Polyposis/MMR | 19 | 91 | 9% | 29% | 26 | ([6](#_ENREF_6)) |
| CRC | MMR (partly) | 22 | 22 | 14% | 23% | 5 | ([7](#_ENREF_7)) |
| CRC | no | 25 | 1058 | 10% | 31% | 408 | ([8](#_ENREF_8)) |
| HBOC | yes | 41 | 20 families | 30% | - | - | ([9](#_ENREF_9)) |
| HBOC | BRCA1/2 | 42 | 141 | 10% | 88% | 428 (in 39 genes) | ([10](#_ENREF_10)) |
| HBOC | no | 68 | 133 | 23% | 9% | 12 | ([11](#_ENREF_11)) |
| HBOC | no | 49 | 26 | 19% | - | 13 | ([12](#_ENREF_12)) |
| HBOC | BRCA1/2 | 25 | 2158 | 4% | 40% | 1197 | ([13](#_ENREF_13)) |
| OC | no | 35 | 117 | 33% | 67% | 145 | ([14](#_ENREF_14)) |
| HBOC, CRC, others | BRCA1/2, MMR genes and other genes | ? | 127 | 7% | 46% | 80 | ([15](#_ENREF_15)) |
| HBOC, CRC, others | BRCA1/2 (n=1097) | 14-21 | 2079 | 8% | 15-26% | - | ([16](#_ENREF_16)) |
| HBOC, CRC | no | 25 | 252,223: | 7% | 30% | - | ([17](#_ENREF_17)) |
|  |  |  | - 100,389 with personal cancer history | 10% |  |  |  |
|  |  |  | - 151,834 unaffected individuals with suspicious family history | 5% |  |  |  |
| HBOC, CRC | BRCA, MMR (partly) | 13-41 | 61 | 7% | 20% | 12 | ([18](#_ENREF_18)) |
| HBOC, CRC, PTC | no | 41 / 55 | 1313 cancer cases | 14% | 42% | - | ([19](#_ENREF_19)) |
|  |  |  | 816 unaffected controls | 2% | - | - |  |
|  |  |  |  |  |  |  |  |
|  |  |  |  |  |  |  |  |
|  |  |  |  |  |  |  |  |
| Abbreviations: CRC = colorectal cancer; HBOC = hereditary breast and ovarian cancer; MMR = mismatch repair; PTC = papillary thyroid cancer; VUS = Variant of uncertain significance | | | | | | | |

**References**

1. Chubb D, Broderick P, Frampton M, Kinnersley B, Sherborne A, Penegar S, et al. Genetic Diagnosis of High-Penetrance Susceptibility for Colorectal Cancer (CRC) Is Achievable for a High Proportion of Familial CRC by Exome Sequencing. J Clin Oncol. 2015.

2. DeRycke MS, Gunawardena S, Balcom JR, Pickart AM, Waltman LA, French AJ, et al. Targeted sequencing of 36 known or putative colorectal cancer susceptibility genes. 2017;5(5):553-69.

3. Hansen MF, Johansen J, Sylvander AE, Bjornevoll I, Talseth-Palmer BA, Lavik LAS, et al. Use of multigene-panel identifies pathogenic variants in several CRC-predisposing genes in patients previously tested for Lynch Syndrome. 2017;92(4):405-14.

4. Kraus C, Rau TT, Lux P, Erlenbach-Wunsch K, Lohr S, Krumbiegel M, et al. Comprehensive screening for mutations associated with colorectal cancer in unselected cases reveals penetrant and nonpenetrant mutations. Int J Cancer. 2014.

5. Pritchard CC, Smith C, Salipante SJ, Lee MK, Thornton AM, Nord AS, et al. ColoSeq provides comprehensive lynch and polyposis syndrome mutational analysis using massively parallel sequencing. J Mol Diagn. 2012;14(4):357-66.

6. Rohlin A, Rambech E, Kvist A, Törngren T, Eiengård F, Lundstam U, et al. Expanding the genotype–phenotype spectrum in hereditary colorectal cancer by gene panel testing. Fam Cancer. 2017;16(2):195-203.

7. Talseth-Palmer BA, Bauer DC, Sjursen W, Evans TJ, McPhillips M, Proietto A, et al. Targeted next-generation sequencing of 22 mismatch repair genes identifies Lynch syndrome families. Cancer Med. 2016;5(5):929-41.

8. Yurgelun MB, Kulke MH, Fuchs CS, Allen BA, Uno H, Hornick JL, et al. Cancer Susceptibility Gene Mutations in Individuals With Colorectal Cancer. J Clin Oncol. 2017;35(10):1086-95.

9. Coppa A, Nicolussi A, D'Inzeo S, Capalbo C, Belardinilli F, Colicchia V, et al. Optimizing the identification of risk-relevant mutations by multigene panel testing in selected hereditary breast/ovarian cancer families. 2018;7(1):46-55.

10. Kurian AW, Hare EE, Mills MA, Kingham KE, McPherson L, Whittemore AS, et al. Clinical evaluation of a multiple-gene sequencing panel for hereditary cancer risk assessment. J Clin Oncol. 2014;32(19):2001-9.

11. Lin PH, Kuo WH, Huang AC, Lu YS, Lin CH, Kuo SH, et al. Multiple gene sequencing for risk assessment in patients with early-onset or familial breast cancer. Oncotarget. 2016;7(7):8310-20.

12. Sung PL, Wen KC, Chen YJ, Chao TC, Tsai YF, Tseng LM, et al. The frequency of cancer predisposition gene mutations in hereditary breast and ovarian cancer patients in Taiwan: From BRCA1/2 to multi-gene panels. 2017;12(9):e0185615.

13. Tung N, Battelli C, Allen B, Kaldate R, Bhatnagar S, Bowles K, et al. Frequency of mutations in individuals with breast cancer referred for BRCA1 and BRCA2 testing using next-generation sequencing with a 25-gene panel. Cancer. 2015;121(1):25-33.

14. Eoh KJ. Detection of Germline Mutations in Patients with Epithelial Ovarian Cancer Using Multi-gene Panels: Beyond BRCA1/2. 2018;50(3):917-25.

15. Frey MK, Kim SH, Bassett RY, Martineau J, Dalton E, Chern JY, et al. Rescreening for genetic mutations using multi-gene panel testing in patients who previously underwent non-informative genetic screening. Gynecol Oncol. 2015;139(2):211-5.

16. LaDuca H, Stuenkel AJ, Dolinsky JS, Keiles S, Tandy S, Pesaran T, et al. Utilization of multigene panels in hereditary cancer predisposition testing: analysis of more than 2,000 patients. Genet Med. 2014;16(11):830-7.

17. Rosenthal ET, Bernhisel R, Brown K, Kidd J, Manley S. Clinical testing with a panel of 25 genes associated with increased cancer risk results in a significant increase in clinically significant findings across a broad range of cancer histories. Cancer Genet. 2017;218-219:58-68.

18. Selkirk CG, Vogel KJ, Newlin AC, Weissman SM, Weiss SM, Wang CH, et al. Cancer genetic testing panels for inherited cancer susceptibility: the clinical experience of a large adult genetics practice. Fam Cancer. 2014;13(4):527-36.

19. Siraj AK, Masoodi T, Bu R, Parvathareddy SK, Al-Badawi IA, Al-Sanea N, et al. Expanding the spectrum of germline variants in cancer. Hum Genet. 2017;136(11-12):1431-44.
